# Dental Almanach

**Published:** 1881-05

**Authors:** 


### DENTAL ALMANACH.

The Dental Almanach for 1881, published by Dr. Adolph Peterman at Frankfort on the Main, is again before us, being now in the fifth year of its issue. This little work is a most valuable one, containing as it does, a list of all the practicing dentists in Germany and Austrian Hungary arranged alphabetically: their places of residence, the institutions and other sources from which they derive their authority to practice, &c. Much new matter has been supplied, making it more complete than any of its predecessors. It still keeps up its rattling thunder against the perfidious practice so long carried on in this country of the sale of bogus diplomas and unearths new cases in a way not calculated to sooth the feelings of those who hold them. We hope that the future of this admirable work may be a continued success.

It may not be amiss to suggest in this connection that an almanach of this character should be gotten up in each of our States. It would go far towards remedying an unmitigated evil. This could be accomplished with comparative ease, were the different State Associations to take the matter in hand; and push it. If there are any who are laboring under the delusion that the bogus diploma business has not been pursued in this State it is time their minds were disabused of the fact. There is at present a bill before the Ohio Legislature to amend the law regulating the practice of medicine, requiring all physicians who have the qualification to practice under the act to register in the office of the Probate Judge of the county wherein they reside and further that all physicians coming from without the state, must submit their diplomas to an authorized body, who shall attest to their genuineness. It is estimated by the author of this bill, that there are over 2000 persons in this State alone practicing under these fraudulent degrees and without any regular course of instruction. The dental profession is infested largely, *we know*, as is shown by the list furnished by "Dr." Buchanan himself.

